# Cr(VI) induces premature senescence through ROS-mediated p53 pathway in L-02 hepatocytes

**DOI:** 10.1038/srep34578

**Published:** 2016-10-04

**Authors:** Yujing Zhang, Yiyuan Zhang, Caigao Zhong, Fang Xiao

**Affiliations:** 1 Department of Health Toxicology, Xiangya School of Public Health, Central South University, Changsha 410078, P.R. China

## Abstract

Hexavalent Chromium [Cr(VI)], which can be found of various uses in industries such as metallurgy and textile dying, can cause a number of human disease including inflammation and cancer. Unlike previous research that focused on Cr(VI)-induced oxidative damage and apoptosis, this study placed emphasis on premature senescence that can be induced by low-dose and long-term Cr(VI) exposure. We found Cr(VI) induced premature senescence in L-02 hepatocytes, as confirmed by increase in senescence associated-β-galactosidase (SA-β-Gal) activity. Cr(VI) stabilized p53 through phosphorylation at Ser15 and increased expression of p53-transcriptional target p21. Mechanism study revealed Cr(VI) targeted and inhibited mitochondrial respiratory chain complex (MRCC) I and II to enhance reactive oxygen species (ROS) production. By applying antioxidant Trolox, we also confirmed that ROS mediated p53 activation. A tetracycline-inducible lentiviral expression system containing shRNA to p53 was used to knockout p53. We found p53 could inhibit pro-survival genes B-cell lymphoma-2 (Bcl-2), myeloid leukemia-1 (Mcl-1) and S phase related cell cycle proteins cyclin-dependent kinase 2 (CDK2), Cyclin E to induce premature senescence, and the functional role of ROS in Cr(VI)-induced premature senescence is depend on p53. The results suggest that Cr(VI) has a role in premature senescence by promoting ROS-dependent p53 activation in L-02 hepatocytes.

Chromium is an extremely important metal which can be found of various uses in industries such as metallurgy and textile dying^1^. Hexavalent Chromium [Cr(VI)] compounds exhibit high mobility in the environment and have been shown to exert toxic effects in most living organisms^2^. In addition, Cr(VI) is a human carcinogen by both the inhalation and oral route of exposure. Senescence, first described by Hayflick and Moorhead in human fibroblast cells in 1961^3^, is characterized by irreversible cell cycle arrest. Cellular senescence is the phenomenon by which normal diploid cells lose the ability to divide with telomere shortening, normally after 60 generations *in vitro*^4^. Senescent cells are flatter and larger than normal proliferating cells with increased β-galactosidase (β-gal) activity, which are different from apoptotic cells^5^. Recently, another type of senescence named premature senescence, which occurs after only few replications cycles and being morphologically and biochemically similar to replicative senescence has been studied a lot^6^. Premature senescence can be triggered by multi-factors including oncogene activation^7^, stress^8^, and xenobiotics exposure^9^.

Reactive oxygen species (ROS), which include free radicals such as superoxide (O_2_) and hydroxyl radicals (HO), are defined as oxygen-containing chemical species with reactive chemical properties^10^. Mitochondrial respiratory chain (MRC) is the most important source of ROS within most cells. ROS produced from MRC complex (MRCC) are of pathological importance in a wide variety of degenerative diseases, aging, and cancer^11^. It has been suggested that Cr(VI) increases cellular ROS levels, such as O_2_^−^ and HO^12^, and our preliminary data showed that Cr(VI) induced an increase of electron leak mainly from the ubiquinone binding sites of complex I and II in isolated rat liver mitochondria.

Senescence is controlled by several tumor suppressor genes such as p53 and retinoblastoma protein (Rb), which lie at the heart of two major senescence pathways, p53-p21^WAF1/CIP1^ and Rb-p16^INK4a^^13^. p53 is a transcriptional activator and repressor that controls the expression of genes that cause cell cycle arrest in response to genomic damage^14^. Tumor suppressor Rb plays a role in the negative control of cell cycle by interacting with transcription factors and remodeling chromatin structures^15^. p53 has a close relation with ROS. p53 activity can be regulated by ROS, and ROS regulate cell fate through p53^16^. In this study, for the first time we focused on the low dose and long-term Cr(VI) exposure on hepatocytes which resulted in premature senescence, and we also did the mechanism studies which revealed that ROS is the key mediator that regulate p53-p21^WAF1/CIP1^ pathway to involve in premature senescence.

## Material and Methods

### Materials

L-02 hepatocyte line was provided by China Center for Type Culture Collection of Wuhan University. 4′6-diamidino- 2 -phenylindole (DAPI), Trolox, and Doxycycline was purchased from Sigma (St. Louis, MO, USA). RPMI-1640 medium, Fetal Bovine Serum (FBS), and Trypsin-EDTA (0.25%) were obtained from Gibco (Gaithersburg, MD, USA). Potassium dichromate (K_2_Cr_2_O_7_) was obtained from Changsha chemical reagents company (changsha, China).

### Cell culture

L-02 hepatocytes were cultured in RPMI-1640 medium supplemented with 10% (vol/vol) FBS, 2 mM L-glutamine, and antibiotics (50 units/ml penicillin and 50 μg/ml streptomycin) at 37 °C under a humidified atmosphere of 5% CO_2_. The medium was changed every other day.

### RNA interference

The primers targeting human p53 short hairpin sequences (Forward (F) primer: gatccccgactccagtggtaatctacttcaagagagtagattaccactggagtctttttta; and the Reverse (R) primer: agcttaaaaaagactccagtggtaatctactctcttgaagtagattaccactggagtcggg) were annealed and cloned into pSUPER vector digested with the BglII and hindIII enzymes. After confirmation of DNA sequences, 293 host cells were transfected with the plasmids to produce viruses. p53 shRNA expression was induced by treatment with 5 μg/ml Doxycycline for 3 days.

### Senescence associated-β-galactosidase (SA-β-gal) staining assay

Cellular senescence was assessed using senescence associated β-galactosidase staining kit (Cell Signaling, Beverly, MA, USA). Briefly, Cells were washed twice with phosphate-buffered saline (PBS, pH 7.4) and then fixed in 0.5% glutaraldehyde for 15 min at room temperature. Then cells were incubated at 37 °C overnight with fresh SA-β-Gal stain solution containing 1 mg/ml of 5-bromo-4-chloro-3-indolyl-beta-D-galactoside (X-Gal). Then the development of blue color (positive stained cells) was checked under microscope.

### Cell cycle analysis by flow cytometry

2 × 10^6^ cells were pelleted by centrifugation and resuspended in 0.5 ml PBS (4 °C), after which 4.5 ml of ethanol (4 °C) was slowly added while vortexing. 2 h later, cells were pelleted and resuspended in PBS. Cells were stained by adding 1 ml PBS containing 50 μg/ml propidium iodide (PI) (Roche, Mannheim, Germany) and 10 μg/ml RNase A (Sigma, St. Louis, MO, USA). Staining was achieved in the dark at room temperature for 30 min. Stained cells were then filtered through 40 μm nylon mesh just prior to running on the flow cytometer. Percentages of cells existing within the various phases (G0/G1, S, G2/M) of the cell cycle were calculated using Cell Quest software (Becton Dickinson, NJ, USA).

### Evaluation of percentage of senescent, apoptotic, and growing cells

Hepatocytes were split into two parts after treatment. One part was stained with SA-β-gal and then was processed to determine the percentage of the senescent cells by counting positive stained cells under microscope. Counts were performed at different random locations and every field was counted three times to determine the mean percentage of positively stained cells. The other part of hepatocytes was double stained with PI and Annexin V (Sigma, St. Louis, MO, USA) and then analyzed by flow cytometry. The percentages of necrotic cells [PI (+), Annexin V (−)], early apoptotic cells [PI (−), Annexin V (+)], and late apoptotic cells [PI (+), Annexin V (+)] were determined. Growing cells were defined as the fraction that was neither senescent nor necrotic nor apoptotic.

### Real-time polymerase chain reaction (RT-PCR) quantitation of gene mRNA expressions

Gene mRNA levels were determined using TaqMan probe-based chemistry (Applied Biosystems, CA, USA), and the relative quantitative values were calculated based on the 2^−ΔΔCt^ method following the manufacturer’s instructions. PCR protocol: initial denaturation for 2 min at 95 °C, 35 cycles of 95 °C for 10 s, 60 °C for 5 s, and 72 °C for 12 s. The sequences of the primers that are being used are as follows: Apo J: F-5′- tggactgttccaccaacaaccc -3′, R-5′ - accgtggtgacccgcagata -3′; CTGF: F-5′- ggcttaccgactggaaga -3′, R-5′ - ccttcttaatgttctcttccagg -3′; FN1: F-5′- cggctgttcctcctccca -3′, R-5′ - ggcaggagatttgttaagaccactgc -3′; Osteonectin: F-5′- caggtttgacactagcatcc -3′, R-5′ - agacctccaggcttct -3′; Cav-1: F-5′- gagggacatctctacaccgt -3′, R-5′ - caatcttgaccacgtcatcgttg -3′; SM22-α: F-5′- tgattctgagcaagctggt -3′, R-5′ - tgccttcaaagaggtcaac -3′; SMP30: F-5′- ctggcaccatggctgaggaaa -3′, R-5′ - ggagatctgtcctgtctgcagg -3′; MRCC I (NDUFS3): F-5′- atgttgcccaaactggtctc -3′, R-5′ - tcactgccttcccagagagt -3′; MRCC II (SDHA): F-5′- agacctgtcctgtggtttgg -3′, R-5′ - agcctttgtccttttgctga -3′; p53: F-5′- ggttgtttcattccgcagtt -3′, R-5′ - ctccaacaggcaaaatggtt -3′.

### Western blotting for protein levels determination

L-02 hepatocytes were lysed using Mammalian Cell Lysis Kit from Sigma-Aldrich (St. Louis, MO, USA). Western blotting was performed with the Western-Breeze Chemiluminescent Immunodetection protocol (Invitrogen, CA, USA). Proteins were separated by electrophoresis on 10% sodium dodecyl sulfate-polyacrylamide gels (SDS-PAGE), and were then transferred to polyvinylidene fluoride (PVDF) membrane by electroelution. The membranes were incubated with primary antibodies overnight at 4 °C following blocking with 4% non-fat milk. Membranes were then incubated for 1 h at room temperature with second antibodies, developed with detection system and then exposed onto films. The quantitative estimation for the Western blotting bands was obtained using Image-pro plus 6.0. software.

The primary antibodies for Apo J (Clusterin-α, sc-32264), CTGF (L20) (SC-14939), and SMP30 (E-11) (sc-390098) were purchased from Santa Cruz Biotechnology (Santa Cruz, CA, USA). Phospho-p53 (Ser 15) (16G8) (#9286), Phospho-p53 (Ser 20) (#9287), Phospho-p53 (Ser 392) (#9281), Bcl-2 (50E3) (#2870), Mcl-1 (#4572), CDK2 (78B2) (#2546), Cyclin E (HE12) (#4129), p21 (12D1) (#2947), Rb (D20) (#9313), and beta-actin (#4967) were purchased from Cell Signaling Technology (Danvers, MA, USA). MRCC I (NDUFS3) (ab110246), MRCC II (SDHA) (ab14715), ATM (Y170) (ab32420), MDM2 (2A10) (ab16895), p53 (PAb 240) (ab26) and p16^INK4a^ (EP1551Y) (ab51243) were purchased from Abcam (Cambridge, MA, USA). FN1 (BA1771) and Cav-1 (BA2440) were purchased from BOSTER (Wuhan, China).

### Determination of IL-1, IL-6 and MMP3 levels

The supernatant of culture medium of both control and Cr(VI)-treated hepatocytes were collected. The senescence-associated secretory phenotype (SASP) related pro-inflammatory cytokines interleukin-1 (IL-1), IL-6, and proteases matrix metalloproteinase-3 (MMP3) in the culture supernatants were determined using ELISA kits purchased from Cusabio Co., Ltd (Wuhan, China). The ELISA was done duplicate according to the manufacturer’s recommendations.

### DAPI stain for Senescence Associated Heterochromatin Foci (SAHF)

To determine SAHF formation, cells were washed with ice-cold PBS and then fixed with 4% ice-cold paraformaldehyde for 30 min. After washing twice with PBS, DNA was visualized by DAPI (1 μg/ml) for 10 min in the dark at 4 °C and then washed twice with PBS. Then the hepatocytes were examined under a microscopy. DNA distribution of control cell nucleus was relatively uniform, and the nuclei were larger and light-stained; the chromatin of senescent cells were condensed and aggregated, and the nuclei were smaller and heavy-stained.

### Measurement of ROS production

Intracellular ROS production was determined by detecting the fluorescent intensity of 2′, 7′-dichlorofluorescein (DCF), the oxidized product of the fluoroprobe 5-(and 6)-chloromethyl-2′, 7′-dichlorodihydrofluorescein diacetate (CM-H2DCFDA, Molecular Probers, USA). Briefly, 2 × 10 cells were collected by centrifugation and then were incubated with 10 μM CM-H2DCFDA in PBS for 40 min at 37 °C in the dark. After incubation, the cells were split into two parts. One part was checked with fluorescence microscope. The other part was measured with flow cytometer with excitation at 488 nm and emission at 535 nm. The amount of ROS production was considered to be directly proportional to the fluorescence intensity.

### Measurement of activities of respiratory chain complexes (MRCC) I- IV

Mitochondria were isolated as previously described^17^ with slight modifications. Cells were washed twice with cold PBS, and resuspended with 5 ml buffer (250 mM sucrose, 20 mM HEPES, 10 mM KCl, 1.5 mM MgCl_2_, 1 mM EDTA, 1 mM EGTA, 1 mM dithiothreitol, 0.1 mM phenylmethylsulfonyl fluoride, pH 7.5). Cells were homogenized and centrifuged twice at 750  × *g* for 10 min. Mitochondria pellets were obtained after centrifugation at 10,000 × *g* for 15 min.

The activities of MRCC were determined using Mitochondrial Respiratory Chain Complexes Activity Assay Kits from Genmed Scientifics (shanghai, China). All assays were performed in a final volume of 1 ml using an UV-9100 spectrophotometer. The activity of MRCC I (Nicotinamide adenine dinucleotide (NADH) CoQ oxidoreductase, expressed as nmol oxidized NADH/min/mg prot) was measured following the oxidation of NADH at 340 nm. The activity of MRCC II (succinate: 2, 6-dichlorophenolindophenol (DCIP) oxireductase, expressed as nmol reduced DCIP/min/mg prot) was measured following the reduction of DCIP at 600 nm. The activity of MRCC III (ubiquinol: cytochrome c (Cyt c) reductase, expressed as nmol reduced Cyt c/min/mg prot) was measured following the reduction of Cyt c at 550 nm. The activity of MRCC IV (Cyt c oxidase, expressed as nmol oxidized Cyt c/min/mg prot) was measured following the oxidation of Cyt c at 550 nm. All measurements were performed in triplicate.

### Statistical analysis

Statistical analysis was performed using SPSS19.0 one-way analysis of variance (ANOVA) to assess the significance of differences between groups. The acceptance level of significance was *p* < 0.05. Results are expressed as mean ± SD of three independent experiments.

## Results

### Cr(VI) induces premature senescence in L-02 hepatocytes

**Figure 1:**
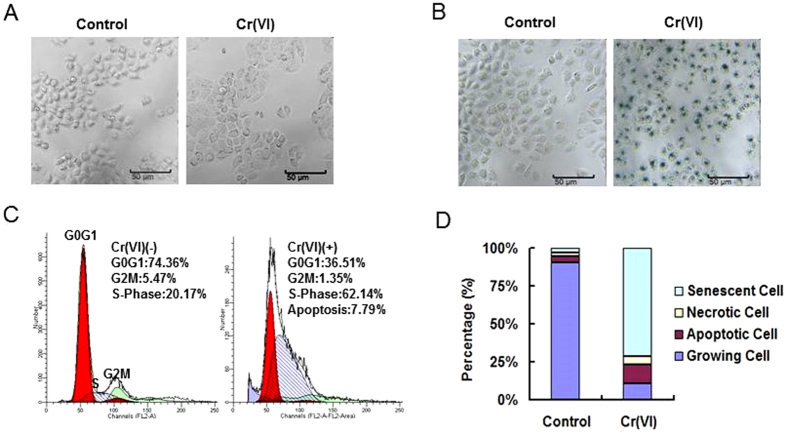
Cr(VI) induced premature senescence in L-02 hepatocytes. Hepatocytes were treated with PBS or 10 nM Cr(VI) twice a week for 24 h for 4 consecutive weeks. (**A**) Morphology analysis revealed that Cr(VI) stimulated cells are flattened, enlarged and more vacuolized (magnification: 40×). (**B**) Hepatocytes were stained with SA-β-Gal activity. Blue stained cells are indicative of cells undergoing senescence. (**C**) Histograms represent the distribution of cells through the cell cycle measured by flow cytometry and analyzed with Cell-Quest software. The percentages of G0/G1 phase (left red peak), G2/M phase (right red peak), S phase (blue oblique line area) and apoptotic cells (blue area left besides G0/G1 peak) are showing on the histograms. (**D**) Percentages of senescent, necrotic, apoptotic and growing cells were examined. The percentage of senescent cells was evaluated as the number of cells expressing SA-β-Gal activity. In parallel, the number of necrotic cells [PI (+), Annexin (−)] and apoptotic cells [PI (−), Annexin (+)] was analyzed by flow cytometry using PI/Annexin V staining. Growing cells are defined as the fraction that is neither apoptotic nor necrotic nor senescent.

L-02 hepatocyte cultures were treated with PBS or 10 nM Cr(VI) twice a week for 24 h for 4 consecutive weeks. Cr(VI) concentration was chosen according to the Cr(VI) values recorded in the blood circulation of exposed workers^18^ and previous study^19^. From the second week of Cr(VI) treatment, cells although viable, appeared growth inhibition and acquired irregular shape which is typical of senescence phenotype. Cells were stained with SA-β-Gal activity every week until the results turned out to be positive. 4 weeks later, Cr(VI) stimulated cells are flattened, enlarged and more vacuolized (Fig. 1A, magnification: 40×). After stained with SA-β-Gal, Cr(VI) treatment group showed large amount of positive stained cells with blue color indicating the occurrence of premature senescence (Fig. 1B). We also examined an additional lower Cr(VI) concentration, 1 nM. The concentration had no effect as treated cells grew similarly to the control cells and did not show SA-β-Gal activity even 8 weeks after the first treatment (data not shown).

The hepatocytes after 4 weeks treatment were also analyzed for cell cycle distribution. In the control group, the percentage of G0/G1, G2/M and S phase were 74.36%, 5.47%, and 20.17%, respectively. A significant S phase arrest was observed in Cr(VI) treatment group which characterized by increased percentage of S phase (62.14%) (Fig. 1C). We further determined the percentage of senescent, growing, apoptotic and necrotic cells in the cultures. As shown in Fig. 1D, more than 90% of the non-treated cells were showing proliferative activity (growing cells). In contract, about 70% of Cr(VI)-treated cells stop proliferating (senescent cells) and only 10% were actively dividing in the presence of the chemical. The percentages of apoptotic cells and necrotic cells showed no significant difference in the two groups.

**Figure 2:**
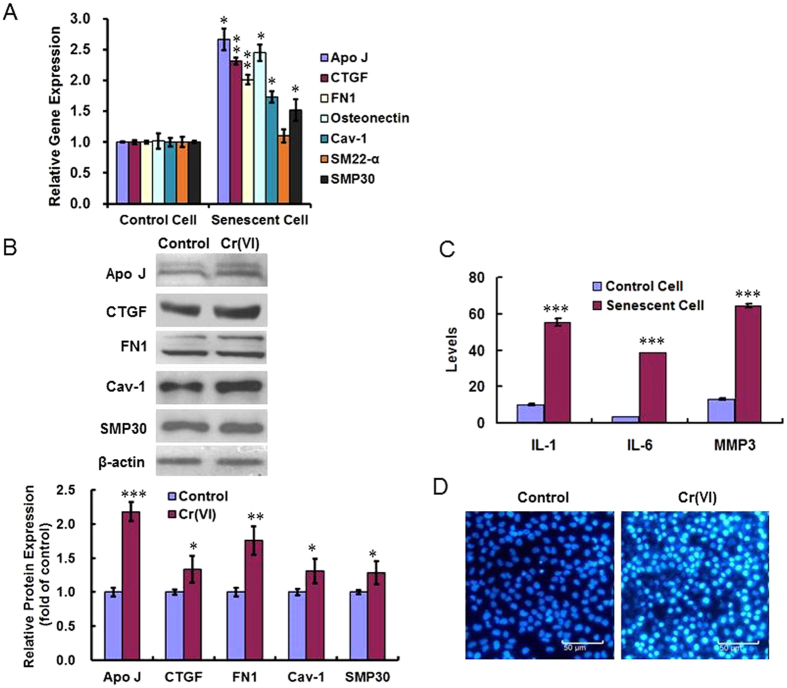
Cr(VI) induced premature senescence in L-02 hepatocytes. (**A**) The senescence biomarkers including Apo J, CTGF, FN, Osteonectin, Caveolin-1, SM22-α, and SMP30 were detected using RT-PCR. *p* values (compared with control): Apo J, 0.0133; CTGF, 0.0012; FN, 0.0033; Osteonectin, 0.0454; Cav-1, 0.0124; SM22-α, 0.4010; SMP30, 0.0496. (**B**) The protein expression levels of the biomarkers were examined using Western blotting methods. The quantitative estimation for all the Western blotting bands was performed using Image-pro plus 6.0. software. *p* values: Apo J, 0.0002; CTGF, 0.0416; FN, 0.0036; Cav-1, 0.0429; SMP30, 0.0453. (**C**) The SASP-related IL-1, IL-6 and MMP3 levels were detected in the supernatant of culture medium of the different treatment groups using ELISA methods. *p* values: IL-1, 0.0000; IL-6, 0.0000; MMP3, 0.0000. (**D**) DAPI stain was used to examine SAHF. *p < 0.05, **p < 0.01, ***p < 0.001, compared with control.

In order to evaluate and study premature senescence, people conclude biomarkers by analyzing pathways and associated mechanisms of premature senescence that caused by various factors. We detected these biomarkers including Apolipoprotein J (Apo J), Connective tissue growth factor (CTGF), Fibronectin (FN), Osteonectin, Caveolin-1 (Cav-1), Smooth muscle protein 22-α (SM22-α), Senecence marker protein-30 (SMP30) using RT-PCR (Fig. 2A) and found that all tested biomarkers showed different degrees of increase except SM22-α. The Western blotting results in Fig. 2B showed the similar changes in protein levels. SASP contain pro-inflammatory cytokines (IL-1, IL-6) and chemokines (IL-8), growth factors (granulocyte-colony stimulating factor [G-CSF], basic fibroblast growth factor [bFGF]), and proteases MMP3^20^. We collected the supernatant of culture medium of the different treatment groups, checked the levels of IL-1, IL-6 and MMP3 using ELISA methods, and found the levels of all these three were increased in the senescent cells (Fig. 2C). In particular, many senescent cells accumulate specialized domains of facultative heterochromatin, called SAHF, which are thought to repress expression of proliferation-promoting genes, thereby contributing to senescence-associated proliferation arrest^21^. When stained with DAPI, senescent cells showed bright punctate DNA foci. The chromatin in these foci appears much more compact than the chromatin in normal interphase growing cells^22^. SAHF is believed to be the main reason for the irreversibility of the aging process. As shown in Fig. 2D, we detected SAHF using DAPI stain and got the positive result in the Cr(VI)-treated cells.

### Cr(VI) induced premature senescence via ROS

**Figure 3:**
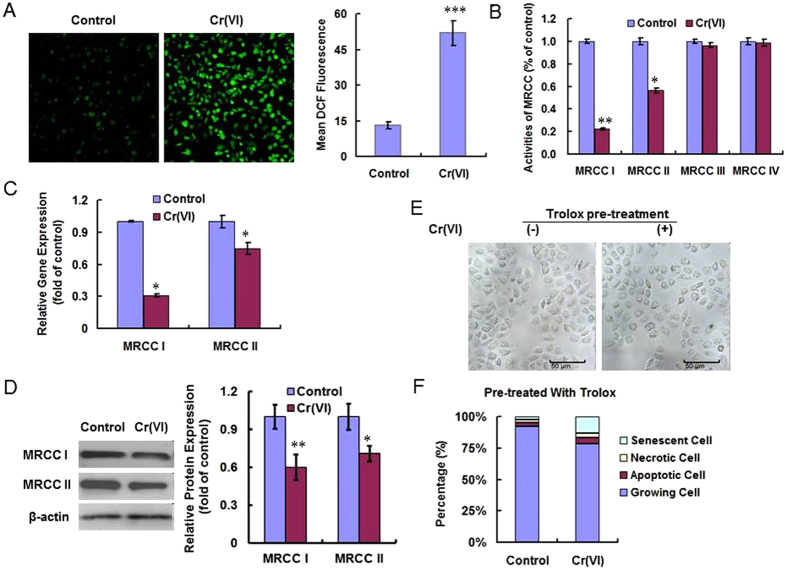
Cr(VI) induced premature senescence via ROS. (**A**) Treated cells were incubated with fluorogenic probe CM-H2DCFDA. Fluorescence was detected by fluorescent microscope (left panel). ROS production was quantitated by flow cytometer (right panel). *p* = 0.0002, compared to control. (**B**) The activities of MRCC I, II, III and IV were measured using kits. All values are expressed as percentage mean ± SD (n = 3) of controls (set to 100%). For MRCC I, *p* = 0.0064; For MRCC II, *p* = 0.0343, compared to control. (**C**) The gene expression levels of MRCC I and II were determined using RT-PCR. For MRCC I, *p* = 0.0213; For MRCC II, *p* = 0.0495, compared to control. (**D**) The protein expression levels of MRCC I and II were determined using Western blotting. For MRCC I, *p* = 0.0078; For MRCC II, *p* = 0.0142, compared to control. (**E**) Hepatocytes were treated with 100 μM Trolox for 1 h prior to Cr(VI) incubation. SA-β-Gal activity was measured 4 weeks later. (**F**) Cells were treated as described in (**E**) and the percentages of senescent, necrotic, apoptotic and growing cells were examined. *p < 0.05, **p < 0.01, ***p < 0.001, compared with control.

L-02 hepatocytes after 4 weeks of treatment were analyzed for ROS production utilizing oxidant-sensitive fluorogenic probe CM-H2DCFDA. As shown in Fig. 3A, Cr(VI) treatment induced much higher level of fluorescence signal, indicating the generation of a large amount of intracellular ROS. Quantitative analysis by flow cytometer showed ROS level was about 4-fold higher in the Cr(VI) treatment group compared with control group. To investigate the mechanism for the elevated ROS level, we determined the activities of MRCC I-IV. The result revealed that the activities of MRCC I and II were significantly decreased in the Cr(VI) exposure group (Fig. 3B). The finding led us to speculate that Cr(VI) could target MRCC I and II, the most susceptible sites to Cr(VI) toxicity to enhance ROS production. We then confirmed Cr(VI) inhibited these two complexes both in gene and protein levels (Fig. 3C,D). SA-β-Gal stain in Fig. 3E showed that pre-treatment with 100 μM Trolox for 1 h prior to Cr(VI) incubation could rescue Cr(VI)-induced premature senescence in L-02 hepatocytes. The alleviation of premature senescence after Trolox pre-treatment was also confirmed by the determination of the percentage of senescent cells. As shown in Fig. 3F, only 10% of the hepatocytes became senescent after Cr(VI) exposure. Then we reached the conclusion that Cr(VI) induced premature senescence via ROS.

### ROS mediated p53 activation in Cr(VI)-induced premature senescence

**Figure 4:**
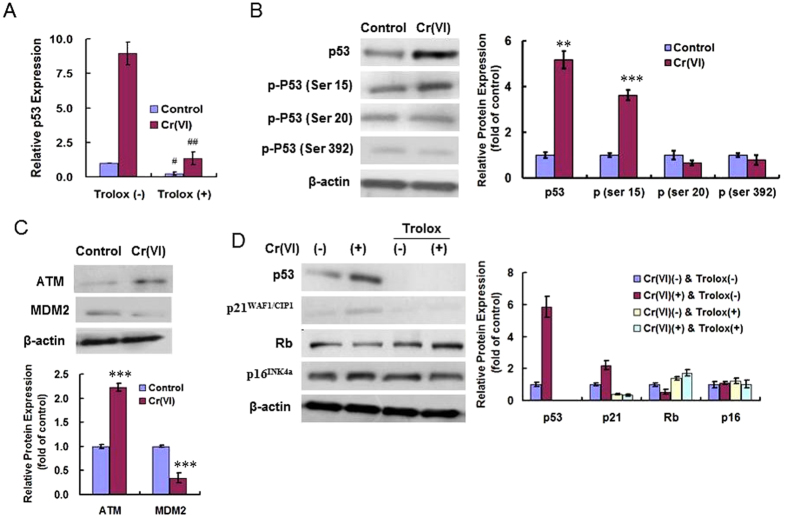
ROS mediated p53 activation in Cr(VI)-induced premature senescence. (**A**) p53 gene expression was determined by real-time PCR in both control and Cr(VI) treatment group with or without Trolox pre-treatment. Data was expressed as 2^−ΔΔCt^ using the p53 expression in the control group [Cr(VI) (−), Trolox (−)] as reference (a 2^−ΔΔCt^ value of 1). *p* values: control, 0.0235; Cr(VI), 0.0077; Trolox (+) compared with Trolox (−). (**B**) Cell lysates were collected for western analysis using specific antibodies. *p* values: p53, 0.0011; p (ser 15), 0.0007. (**C**) The protein levels of ATM and MDM2 were examined using Western blotting. *p* values: ATM, 0.0000; MDM2, 0.0004. (**D**) Western blotting was done to analyze senescence pathways. ^#^*p* < 0.05, ^##^*p* < 0.01, Trolox (+) compared with Trolox (−). *p < 0.05, **p < 0.01, ***p < 0.001, compared with control.

We determined p53 gene expression by real-time PCR. As shown in Fig. 4A, the expression of p53 was about 9-fold higher after Cr(VI) treatment compared with control, indicating the activation of p53 in the senescent cells. Trolox pre-treatment significantly decreased p53 expression levels in both the control and the Cr(VI) exposure group, revealing that ROS could transcriptionally regulate p53. It is generally believed that post-translational modifications like phosphorylation play a role in stabilizing p53 protein. After Cr(VI) treatment, p53 expression significantly increased in the senescent cells. Phosphorylation of p53 at Ser 15 exhibited a clear increase. In contrast, phosphorylation of N-terminal residue Ser 20 and C-terminal residue Ser 392 showed no change in response to stimulation with Cr(VI) (Fig. 4B). Murine double minute 2 (MDM2) is known to be the most important negative regulator of p53^23^, and it has been confirmed that ataxia telangiectasia mutated (ATM) phosphorylation of MDM2 is likely to be the key step in causing p53 stabilization^24^. We examined ATM and MDM2 protein expressions and found that ATM showed increased protein level while MDM2 showed decreased protein level in the senescent cells induced by Cr(VI) (Fig. 4C). Western blotting for senescence pathway analysis in Fig. 4D revealed that p53-p21^WAF1/CIP1^ pathway, but not Rb-p16^INK4a^ pathway was involved in Cr(VI)-induced premature senescence. p53 and p21^WAF1/CIP1^ was up-regulated after Cr(VI) exposure, while Rb and p16^INK4a^ showed no significantly change compared with control group. Trolox pre-treatment resulted in almost undetectable p53 expression in both the control and Cr(VI) treatment group, indicating the role of ROS played in regulating p53 expression. Trolox also blocked the induction of p21^WAF1/CIP1^ in Cr(VI) treatment group, but did not alter the expressions of Rb and p16^INK4a^.

### Knocking-out p53 by siRNA blocks the induction of premature senescence in Cr(VI)-treated hepatocytes

**Figure 5:**
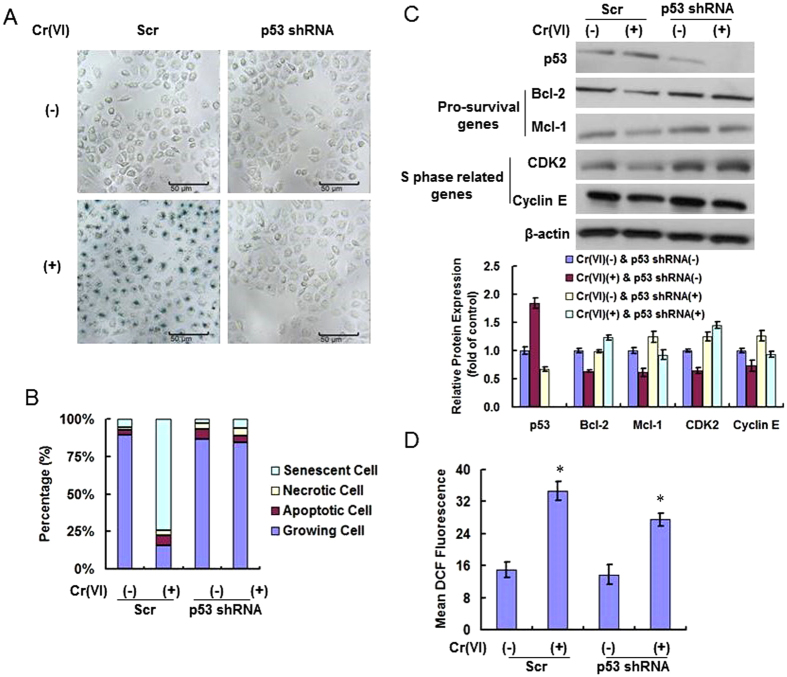
Knocking-out p53 by siRNA blocks the induction of premature senescence in Cr(VI)-treated hepatocytes. A Doxcycline-inducible lentiviral expression system containing shRNA to p53 was used to knockout p53. The non-target scramble (Scr)- and p53 shRNA- transfected hepatocytes were treated with PBS or 10 nM Cr(VI) twice a week for 24 h for 4 consecutive weeks. (**A**) SA-β-Gal activity was measured to explore the effect of p53 on Cr(VI)-induced senescence. (**B**) Percentages of senescent, necrotic, apoptotic and growing cells were examined. (**C**) Western blotting was done to analyze the protein levels of p53, pro-survival genes and S phase related proteins. (**D**) ROS production was quantitated by flow cytometer. For the Scr groups, *p* = 0.0166; for p53 sh RNA groups, *p* = 0.0291. The values were expressed as mean ± SD of 3 independent experiments. *p < 0.05, compared with control.

We further checked if Cr(VI)-induced premature senescence was completely depend on p53 function. A Doxcycline-inducible lentiviral expression system containing shRNA to p53 was used to knockout p53 in hepatocytes. The non-target scramble (Scr)-transfected and p53 shRNA-transfected hepatocytes were treated with PBS or 10 nM Cr(VI) twice a week for 24 h for 4 consecutive weeks. Cr(VI) induced senescence in Scr cells but not in p53 shRNA cells, indicating the absence of p53 blocked the induction of senescence (Fig. 5A). This was further confirmed by checking the percentage of senescent cells in the cultures. As shown in Fig. 5B, about 75% of Scr cells in the Cr(VI) treatment group were showing SA-β-Gal activity (senescent cells). In contrast, more than 80% of p53 shRNA cells were actively dividing in the presence of the chemical, and the percentage of apoptotic cells showed a slight increase. Western blots confirmed the knockout of p53 proteins in L-02 hepatocytes after transfection of p53 shRNA (Fig. 5C). We further checked the pro-survival genes [B-cell lymphoma-2 (Bcl-2), myeloid leukemia-1 (Mcl-1)] and S phase related proteins [cyclin-dependent kinase 2 (CDK2), Cyclin E]. In the Scr cells, Bcl-2 and Mcl-1 were decreased in the Cr(VI) treatment groups, indicating the inhibition of cell proliferation. In p53 shRNA cells, the expressions of the pro-survival genes were increased, and not altered after Cr(VI) exposure, indicating that p53 could regulate pro-survival genes. The same results were observed for CDK2 and Cyclin E. The decreased expression of CDK2 and Cyclin E indicating the disturbance of S phase progression and p53 could regulate S phase related proteins to cause S phase arrest in senescent cells. We also checked the ROS levels in the above cultures. Cr(VI) induced ROS accumulation in both the Scr and p53 shRNA cells (Fig. 5D).

## Discussion

Cellular senescence is a state of permanent cell cycle arrest contributing to tissue aging and has been considered in recent years as an intrinsic barrier against tumorigenesis^25^. In cell cultures, typically, cell cycle arrest lead to senescence but cell cycle arrest is not senescence^26^. Occupational exposure to Cr(VI) generally occurs by low-dose and long-term inhalation and dermal contact. SA-β-gal activity is the most extensively used biomarker for senescence because of the simplicity of the assay method and its specificity for senescent cells. Some thinks that the identification of senescence rests solely on the detection of SA-β-gal activity. In fact, it is reported that β-gal activity can be detected in cells in various non-senescent states such as extended incubation at high density, and there are conflicting reports regarding the presence of SA-β-gal activity in aged tissues^27^,^28^. Thus, the suitability of SA-β-gal as a marker of senescence has been challenged^28^,^29^,^30^. In our present work we also examined other indicators of senescence including some biomarkers (such as Apo J, CTGF, and FN1), SASP and SAHF to confirm “true” senescence occurred. Apo J is an oxidative stress-responsive gene and is often up-regulated following the exposure of the senescence inducers^31^. Apo J is not able to induce senescence, but rather a secondary consequence of the senescence phenotype. CTGF, a useful marker for the identification of senescence, is an age-associated protein that functioned by transforming growth factor- β (TGF-β) pathway^32^. FN expression can be markedly increased in senescent cells, which has been found correlates closely with the increasing size of senescent cells^33^. Cav-1, which related to the activation of p53/p21^WAF1/CIP1^ and G0/G1 phase arrest in senescent cells, may function as a mediator in premature senescence to suppress tumor formation^34^. Most of previous researches associated with Cr(VI)-induced cytotoxicity were focused on apoptosis and the related mechanisms. In our previous published work we demonstrated that Cr(VI) targeted and inhibited MRCCs to induce ROS accumulation and then caused apoptosis. MRCC I and III showed significant decreased activities when the hepatocytes were treated with 20 or 40 μM Cr(VI) for 12 h^35^, while MRCC I and II activities were inhibited when 4–32 μM Cr(VI) were used to treat the cells for 48 h^36^. Our present study revealed that long-term and low-dosage exposure of Cr(VI) induced premature senescence by inhibiting MRCC I and II and then leading ROS accumulation. Although Cr(VI)-induced inhibition of MRCCs depend on Cr(VI) treatment time and concentration, we concluded that MRCC I was the one that most likely to be affected. NADH:ubiquinone oxidoreductase (complex I) is an inner mitochondrial membrane-bound multi-subunit enzyme complex. Complex I consists of at least 45 subunits of which 38 subunits are encoded by nuclear genome and 7 are encoded by the mitochondrial genome^37^,^38^. The present study focused on the subunit NDUFS3 because our preliminary data of gene chip result revealed that NDUFS3 was significantly down-regulated by Cr(VI) while there were no apparent change in other genes. It is known that mutations in NDUFS3 gene are associated with MRCC I deficiency, which is the most common enzymatic defect of the oxidative phosphorylation disorders^39^.

MDM2, which promotes p53 degradation by forming a stable complex through MDM2 and p53 N-terminal domains, is a negative regulator of p53^40^. It has been confirmed that ATM phosphorylation of MDM2 is likely to be the key step in causing p53 stabilization^24^. Our current result revealed that ATM and MDM2 may associated with Cr(VI)-induced stabilization of p53 and we are still focusing on the related implied mechanisms. During the induction of senescence, p53 transactivates p21^WAF1/CIP1^ gene, which is the only known mediator of its pro-senescent function, and p21^WAF1/CIP1^ protein binds to and inhibits CDK2 and Cyclin E, which lead to S phase cell cycle arrest and senescence^41^. Bcl-2 and Mcl-1 are Bcl-2 family members, they are known as pro-survival genes that could indicate cell proliferative activity^42^. Bcl-2 promotor contains a p53-negative response element, which may account for p53-mediated transrepression of Bcl-2^43^.

We knocked out p53 in L-02 hepatocytes to explore p53 function in premature senescence. Although we have confirmed that Cr(VI)-induced premature senescence in hepatocytes is depend on ROS-p53, we are surprised that other pathways were not involved in ROS-mediated premature senescence, as it has been reported that ROS can activate mammalian target of rapamycin (mTOR), the activation of which can contribute to and be essential for certain types of senescence^44^. It has also been suggested that ROS can activate p38 MAP kinase (MAPK) and extracelluar regulated protein kinase (ERK), which in turn activate Rb-p16^INK4a^ pathway to induce senescence^45^. Surprisingly, we did not observe the involvement of Rb-p16^INK4a^ pathway in Cr(VI)-induced premature senescence. A lot of questions remain to be answered, and we still have a long way to go to fully understand Cr(VI)-induced premature senescence.

Exposure to Cr(VI) has been known to be associated with induction of cancer in humans^46^. Despite the adverse effect on physiological activities of cells, Cr(VI)-induced premature senescence can be also viewed as an intrinsic barrier against tumorigenesis. After exposed to Cr(VI), cells may become senescent, but a small subset of cells can bypass premature senescence to become survivors, that is why we should also focus on the “unaffected” cells. The survivors will either restore normal function or become pre-tumor cells with damaged DNA, and senescence bypass appears to be an important step in the development of cancer. Indeed, lots of findings raise the possibility that senescent cells create a special environment that inhibits cell proliferative activity, thereby functioning as tumor suppressive mechanism. It is still not clear after Cr(VI) exposure, how the senescent cells alter the surrounding microenvironment and what impact these alterations will have on tumorigenesis. Next we will focus on the correlation between Cr(VI)-induced senescence and tumorigenesis.

## Additional Information

**How to cite this article**: Zhang, Y. *et al*. Cr(VI) induces premature senescence through ROS-mediated p53 pathway in L-02 hepatocytes. *Sci. Rep.* **6**, 34578; doi: 10.1038/srep34578 (2016).
